# Training Enhances the Interference of Numerosity on Duration Judgement

**DOI:** 10.1371/journal.pone.0054098

**Published:** 2013-01-11

**Authors:** Amir Homayoun Javadi, Clarisse Aichelburg

**Affiliations:** 1 Section of Systems Neuroscience, Department of Psychiatry and Psychotherapy, Technische Universität Dresden, Dresden, Germany; 2 Institute of Cognitive Neuroscience (ICN), University College London (UCL), London, United Kingdom; 3 Division of Psychology and Language Sciences, University College London (UCL), London, United Kingdom; Tokai University, Japan

## Abstract

The interference of magnitudes in different dimensions has been demonstrated previously, but the effect of training in one dimension on judgment of another has yet to be examined. The present study aimed to investigate the effect of training in numerosity judgment on judgment of duration. 32 participants took part in two sessions, 12 days apart, and had to judge which of two successive sets of items was presented longer. Half of the participants (training group) were additionally trained in 11 sessions to judge which one of the two successive sets of items was more numerous. It was found that the participants in the training group became more prone to the interference of numerosity on judging duration after training, when compared to the control group. Thus, being trained to more easily perceive the difference in number of items in the two sets affected the perception of duration. On the 3-month follow up session, no effect was found with 20 participants (n = 10 for each group). These findings indicate that the interference of magnitudes in different dimensions can be modulated by training. We discuss that this modulatory effect might be due to neural changes in shared brain regions between interfering magnitudes and/or is mediated by higher levels of perception.

## Introduction

Perception of magnitudes in different dimensions, e.g. quantity, length, duration, speed, brightness, weight etc., is ubiquitous across most animal species and all phases of human life [1]–[5]. The ability to process magnitudes in one dimension or another is developed early in life. Even pre-linguistic infants, being only 4.5 to 8 months of age, show some numerical competence [6], [7]. They are able to discriminate between stimuli consisting of 1, 2 and 3 items and they can even perform basic arithmetic, such as 1+1 = 2 as well as 3–1 = 2 [8], [9]. These processing abilities continue to develop throughout adolescence and adulthood due to training and environmental changes [10].

Numerous studies have shown interference and association of one dimension with another, suggesting possible commonality(s) on the neural and/or perceptual level. The ability to record numbers and time has been demonstrated in animals by Meck & Church [11]. They showed that the same mechanism is involved for counting and timing. Additionally, electrophysiological and neuroimaging studies have identified a common network of brain regions processing numbers and time [12]. This evidence has been interpreted as indicative of a common neuronal structure for magnitudes. Based on previous findings of interactions between magnitudes, Walsh [13], [14] proposed that magnitudes are interconnected within shared brain areas, which he referred to as a generalised magnitude system, proposing ‘A theory of magnitude’ (ATOM). According to this theory, the shared properties of dimensions such as space, time and quantity and their close cooperation in interaction with spatial and temporal structure of the external world, are suggestive of a common brain area, namely the parietal cortex. Therefore, magnitudes can sometimes interfere with each other leading to misperceptions of one dimension or another, as investigated by this study.

An intuitive ‘more A-more B’ mapping between different dimensions has been proposed by Stavy & Tirosh [15], suggesting, for example, that the bigger a train is, the faster it is perceived to be. Horne and Turnbull, as well as Lechelt and Nelson showed that an increasing or decreasing number of lights [16] or a set of serially presented lights [17] is perceived more numerous if presented for a longer duration. Furthermore, Xuan, Zhang, He, & Chen [18] found that irrelevant magnitude information, such as size, luminance, and numerosity, can affect temporal judgements. Using Stroop-like paradigms, they found that stimuli with larger magnitudes in nontemporal dimensions were perceived as being presented longer. Another study by Oliveri, Vicario, & Salerno [19], who used a time estimation task, found that high digits lead to an overestimation, whereas low digits lead to an underestimation of perceived duration. Thus, a temporal duration judgement can be biased by a number’s magnitude. Dormal et al [20] had subjects compare two successive series of flashing dots, and found that numerical cues interfered with the duration processing, but temporal cues did not interfere with numerosity processing. A recent study by Javadi and Aichelburg [21], in contrast, identified a reciprocal relation between judgement of duration and numerosity. Their results showed that a set of items was perceived as being more numerous when it was presented for a longer duration, and vice versa, i.e. a set was perceived as being presented longer, when it contained more items.

Considering the inconsistency in the literature, the relationship between these two dimensions necessitated further investigation using a method that allows modulating the perception of one dimension to examine whether this also modulates the perception of another dimension.

To the best of our knowledge, the effect of training on the interference of two magnitudes has not yet been examined that may provide valuable insights into the relation of two dimensions. All of the mentioned studies were performed on participants with no prior training on judgement of the certain dimension of interest. The present study aimed to investigate the effect of training in one dimension (numerosity) on judgement of another dimension (duration). All participants took part in two sessions. They had to judge which of two successive sets of items was presented longer. In between these two sessions, half of the participants (training group) were additionally trained to judge which one of the two successive sets of items was more numerous. We anticipated that training would facilitate participants to perceive the changes of the interfering dimension more readily, therefore leading to increased interference, when compared to the untrained, control condition. Additionally, we ran a follow-up session after 3 months to investigate the lasting effects of training on the interference of numerosity on judgement of duration.

## Methods

### Participants

**Figure 1:**
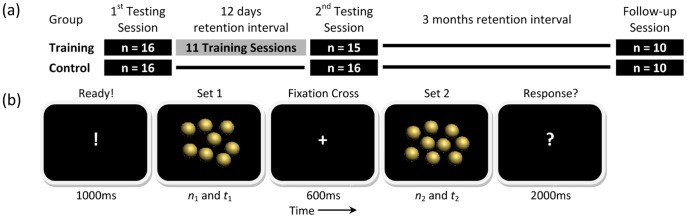
Design of the study. (a) Procedure of the study. (b) Procedure of a trial in testing and training sessions. Refer to the text for description of *n* and *t.*

Thirty-two (17 females, 18–20 years old) subjects took part divided into two experimental groups: training (n = 16) and control (n = 16). All the participants were healthy with no history of neurological or psychiatric disorder, right handed, with normal or corrected-to-normal vision, and were naive to the purpose of the study. One participant in the training group dropped out due to illness during the training period. After 3 months, all 31 participants were invited to a follow-up testing session to study the persisting effects of the training. Twenty of them (10 in each group) took part in this testing session. Figure 1(a) shows the design of the experiment and the order of the three sessions. All participants gave a written, informed consent in accordance with the Declaration of Helsinki. The study protocol was approved by the ethics committee of University College London (UCL).

### Apparatus

Experiments were run on desktop computers with a 17-inch CRT monitor and 75 Hz refresh rate with a resolution of 1024×768 pixels. The monitor was placed 53cm from the participants’ eyes. Stimuli presentation and the recording of response time were accomplished using MATLAB (v7.5; MathWorks Company) and the Psychtoolbox v3 [22], [23]. Data analyses were performed using Palamedes toolbox for MATLAB [24] and SPSS (v17.0; LEAD Technologies, Inc.). Responses were made on a conventional computer keyboard using index and middle fingers of the participants’ right hand.

### Stimuli

Stimuli were sets of items consisting of the image of a synthetic ball placed in random locations within a 25.32×19.12 visual degrees virtual rectangle at the centre of the monitor on a black background. The items were a solid yellow sphere with a mild shading created by 3DS Max (Autodesk) and 1.61×1.61 visual degrees. In order to avoid overlap and to assure distance between items, they were set apart by at least 5/2 of their radius (measured from their centres).

### Design

The study adopted a mixed-factor design with three *testing* sessions (1^st^, 2^nd^ and follow-up testing sessions). The retention interval between the 1^st^ and 2^nd^ testing sessions was 12 days and 3 months between the 2^nd^ and the follow-up testing session. Participants were randomly assigned to either group: training or control. All participants took part in two sessions, on Saturday and Friday in the time span of two weeks (12 days apart). During these 12 days, half of the participants (training group) were additionally trained for 11 sessions. *Training* sessions began on the Sunday after the first testing session with only one training session on the following weekend. Figure 1(a) shows the timing of the 3 testing and 11 training sessions.

In the testing sessions, participants had to compare the duration of presentation in two consecutively shown sets of items and select the set that was presented longer (duration judgement), whereas during the training sessions they had to compare the number of items in the two consecutive sets and select the more numerous one (numerosity judgement). Two independent variables, namely the duration of presentation of each set (*t_1_* and *t_2_*) and the number of items in each set (*n_1_* and *n_2_*), were modified. Trials in the testing sessions were either ‘veridical’ or ‘phantom’. *Veridical* trials were the trials in which the number of items in the two sets were the same, but the durations of presentation were different (*n_1_* =  *n_2_* = 28, *t_1_* ≠ *t_2_* and *t* ∈ {53 ms, 66 ms, 80 ms, 93 ms, 106 ms}). *Phantom* trials, on the other hand, were the trials in which, the number of items in the two sets were different, while the durations of the presentation were identical (*n_1_* ≠ *n_2_*, *t_1_* =  *t_2_* = 80ms and *n* ∈ {28, 31, 34, 37, 40}). The values for *n* and *t* were selected based on Javadi and Aichelburg [21].

Training sessions composed only of trials in which the number of items in the two sets were different while keeping the durations of presentation constant (*n_1_* ≠ *n_2_*, *t_1_* =  *t_2_* = 80ms and *n* ∈ {28, 31, 34, 37, 40}).

Javadi and Aichelburg [21] showed that total occupied area, size, and density do not affect the judgement of the participants in this task, considering the current presentation magnitudes of duration and numerosity. Therefore, we did not control for the total occupancy of the items to keep the stimuli as simple as possible not to distract the participant with complexity of the stimuli.

### Procedure

Testing sessions were composed of eight blocks of 80 trials (8 repetitions per absolute value of difference level |*n_2_*– *n_1_*| and |*t_2_*– *t_1_*|, see below), resulting in 320 veridical and 320 phantom trials in total. Although the trials in which both *n_2_*– *n_1_* = 0 and *t_2_*– *t_1_* = 0 were the same in between veridical and phantom trials, we included separate trials for the two types of trials to keep the number of samples in all conditions equal. Training sessions did not include any veridical trial, therefore the total number of trials were half of the trials in testing sessions. The procedure of one trial is shown in Figure 1(b).

After each block, feedback was given based on the participant’s performance on the veridical trials. Participants were instructed to respond as accurately and as quickly as possible, within the response period. Participants were also asked to keep their gaze point at the centre of the monitor at all times.

### Statistical Analysis

Performance and response times were recorded. Performance refers to the percentage of selecting the first set. A logistic psychometric function, P(*k*) = 100/(1+ exp(-β (*k* – α))), was fitted to performance and mean response time over *k* = *t*_1_ − *t*_2_ (9 levels) for veridical trials and *k* = *n*_1_ − *n*_2_ (9 levels) for phantom trials for each participant and for each testing session. Two free parameters were used for curve fitting: α is the point of maximum growth, indicating the *point of subjective equality (PSE)* and β is the growth rate, indicating the *sensitivity* to different magnitudes. The logistic function has been widely used to describe psychometric functions. One of its main advantages is independency of the two parameters of PSE and sensitivity.

Four separate 2×2 mixed-factor analysis of variances (ANOVA) with testing session number (1^st^/2^nd^ session) as within subject factor and group (training/control) as between subject factor, were conducted on the two dependent variables (α and β) for phantom and veridical conditions. Post-hoc Bonferroni corrected two-tailed independent sample *t*-tests were run to compare the performance of the two groups in the two testing sessions.

Additionally the performance and response time of the participants in the follow-up session in the two groups were compared using two-tailed independent sample *t*-test. As about one-third of the whole sample was unable to participate in the follow-up session, we did not run a large 3×2 mixed-factor ANOVA with sessions (1^st^/2^nd^/follow-up session) as within subject factor and group as between subject factor.

The performance of the participants throughout the training sessions was also analysed. Two two-tailed paired-samples *t*-tests were conducted on the performance accuracy and response time of the participants in the first and the last training session. Data was tested for normality.

## Results

**Figure 2:**
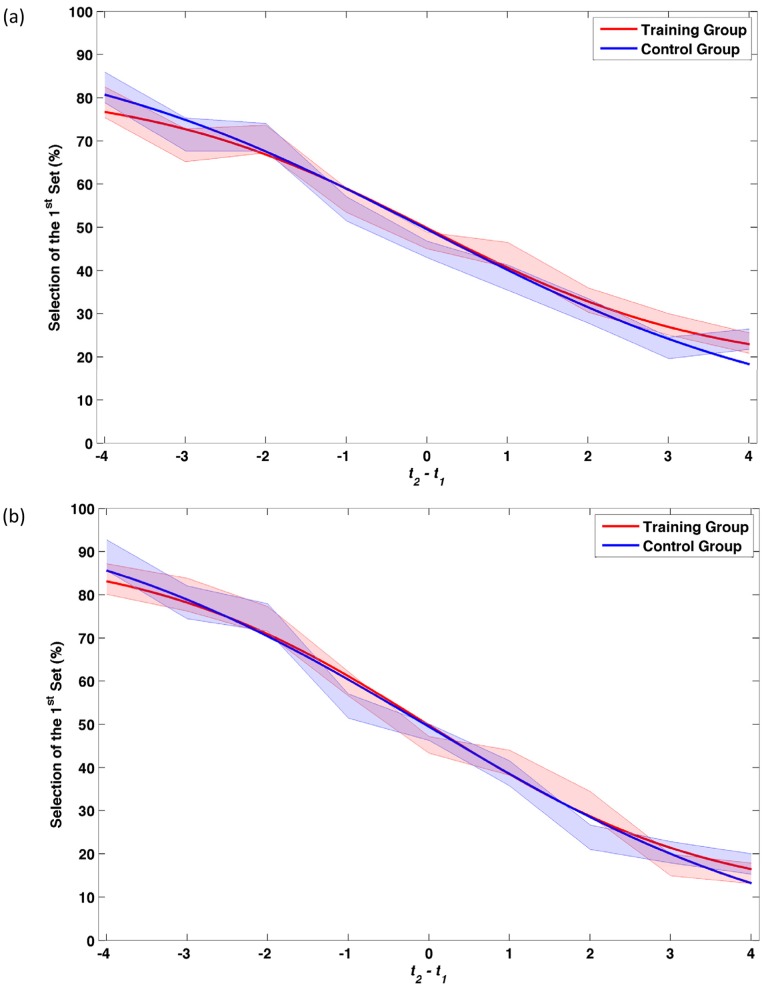
Percentage of selection of the 1^st^ set for *veridical* trials (*n_1_* =  *n_2_* and *t_1_* ≠ *t_2_*). (a) 1^st^ testing session, (b) 2^nd^ testing session. The shaded areas represent one SD around the mean.

A 2×2 mixed-factor ANOVA with group and session number as independent factors and β for veridical trials as dependent factor showed no significant difference in any of the comparisons (*F* <1). Figure 2 shows the performance of the participants for veridical trials.

**Figure 3:**
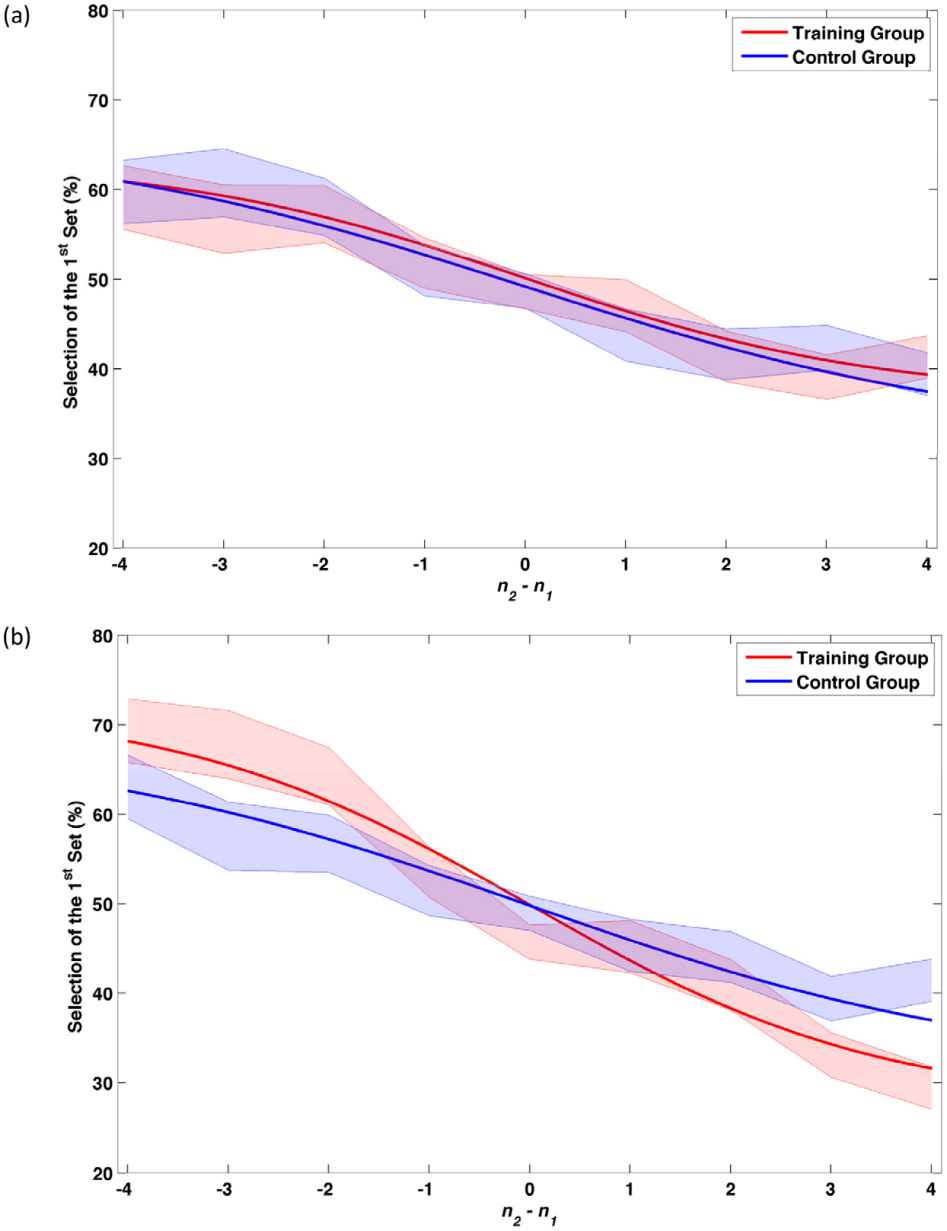
Percentage of selection of the 1^st^ set for *phantom* trials (*n_1_* ≠ *n_2_* and *t_1_* =  *t_2_*). (a) 1^st^ testing session, (b) 2^nd^ testing session. The shaded areas represent one SD around the mean.

Correspondingly, for phantom trials, a 2×2 mixed-factor ANOVA on β showed no significant effect of session (*F*(1, 29) = 2.482, *p* = 0.13), no significant effect of group (*F*(1, 29) = 2.87, *p* = 0.10) but a significant effect of interaction (F(1, 29) = 6.06, *p* = 0.02). Figure 3 shows the performance of the participants for phantom trials.

Post-hoc Bonferroni corrected independent-sample *t*-tests on the β values of the two groups in the two sessions showed no significant difference for the first session (*t*(29) = 0.41, *p* = 0.68) but a significant difference for the second session (*t*(29) = 2.68, *p* = 0.01).

α values were subjected to similar 2×2 mixed-factor ANOVAs for veridical and phantom conditions. These analyses showed no significant difference in any of the comparisons (*F* <1). Table 1 shows the mean and standard deviation (SD) of α and β for different conditions and groups over the two testing sessions.

**Table 1:** The mean and SD (in parentheses) for α (point of subjective equality) and β (sensitivity) for veridical and phantom trials over the 1^st^ and 2^nd^ testing sessions split over the groups.

		Veridical		Phantom	
	Group	Testing 1	Testing 2	Testing 1	Testing 2
**α**	**Control**	0.11 (1.06)	−0.03 (0.93)	1.20 (0.89)	1.08 (0.78)
	**Training**	0.74 (1.96)	0.60 (1.68)	0.87 (1.29)	1.40 (0.91)
**β**	**Control**	−0.59 (0.13)	−0.73 (0.13)	−0.20 (0.02)	−0.22 (0.02)
	**Training**	−0.63 (0.33)	−0.83 (0.40)	−0.27 (0.25)	−0.39 (0.12)

Similarly the response times were analysed using 2×2 mixed-factor ANOVAs. None of the comparisons were significant (*F* <1). Table 2 shows the mean and standard deviation (SD) of response times for different conditions and groups over the two testing sessions.

**Table 2:** The mean and SD (in parentheses) of response times (s) for veridical and phantom trials over the 1^st^ and 2^nd^ testing sessions split over the groups.

	Veridical		Phantom	
Group	Testing 1	Testing 2	Testing 1	Testing 2
**Control**	0.59 (0.30)	0.53 (0.29)	0.59 (0.29)	0.54 (0.29)
**Training**	0.61 (0.31)	0.54 (0.28)	0.61 (0.31)	0.53 (0.28)

Performance of the participants in the follow-up session were also subjected to two independent sample *t*-tests for α and β. This analysis showed no significant difference for α (*t*(18) = 0.58, *p* = 0.57) and no significant difference for β (*t*(18) = 1.63, *p* = 0.12). Similarly the response times were analysed. None of the comparisons were significant (*t* <1).

**Figure 4:**
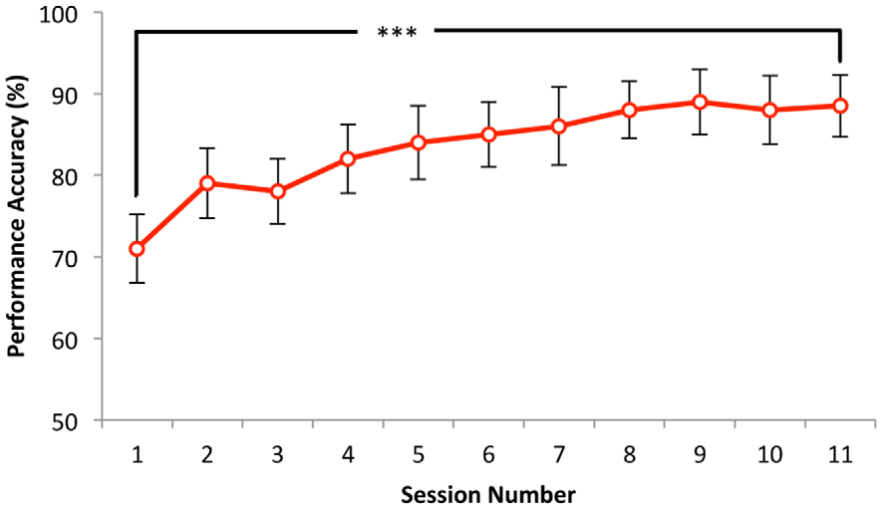
Performance accuracy of the training group on numerosity judgement task during 11 training sessions. *** *p*<0.001.

Percentage performance accuracy of the participants in the training group during the training sessions was also analysed. A paired *t*-test comparing the accuracy of the participants in the first training session and the last training session (11^th^ session) showed a highly significant difference (*t*(14) = 4.32, *p*<0.001), Figure 4.

## Discussion

Judgement of numerosity and duration has been shown to interfere. The effect of training in one dimension (numerosity) on judgement of another dimension (duration) was investigated in the present study. The main question under investigation was if perceiving differences in numerosity was enhanced via training, would this affect the interference of numerosity on judgement of duration? The results showed that participants in the training group became more prone to the interference of numerosity on judgement of duration after training when compared to participants in the control group, as assessed by sensitivity parameter (‘β’). However, there was no significant effect in the follow-up session.

No significant difference in point of subjective equality (PSE) (‘α’) assessed by two separate 2×2 ANOVA on veridical and phantom trials shows that participants in the two groups and over the two testing sessions were not biased differently towards either the first and second sets.

PSE and sensitivity (‘β’) parameters of the fitted curves over response times were also subjected to two similar ANOVAs. No effect was significant, although one might expect to have faster response times on the second session of the training group as they underwent 11 sessions of training with similar response procedure. We speculate that it is because the tasks were orthogonal, i.e. judgement of duration on testing sessions and numerosity on training sessions. A closer look at response times over blocks in each testing session showed that participants’ response time dropped abruptly from the 1^st^ block to the 2^nd^ and remained fairly stable thereafter. Table 3 summarises reaction times for the first two and last blocks (1^st^, 2^nd^ and 8^th^). This shows that participants in both groups achieved the shortest response time over the 1^st^ block of each session.

**Table 3:** The mean and SD (in parentheses) of response times (s) for veridical and phantom trials split over the groups for the 2^nd^ session.

	Veridical		Phantom	
Block	Control	Training	Control	Training
**1^st^**	0.59 (0.27)	0.64 (0.29)	0.58 (0.26)	0.60 (0.28)
**2^nd^**	0.53 (0.26)	0.56 (0.27)	0.54 (0.30)	0.54 (0.26)
**8^th^**	0.51 (0.28)	0.54 (0.26)	0.52 (0.29)	0.53 (0.25)

Behavioural studies aimed to investigate the association of numerosity and duration perception. Xuan et al. [18] found that stimuli with larger magnitudes in nontemporal dimensions were perceived as being presented longer. Furthermore, Oliveri et al. [19] showed a temporal duration judgement can be biased by a number’s magnitude. Dormal et al. [20] used flashing dots in a series and had subjects compare two successive series, and found that numerical cues interfered with the duration processing. Droit-Volet, Clément and Fayol [25], in a study on children aged 5 and 8 years old, showed that in a temporal bisection task, number interfered with temporal performance (more strongly for 5-year-old children). Recently, a reciprocal relation between duration and numerosity has been identified [21]. In accordance with these findings, Stavy and Tirosh [15] have suggested an intuitive ‘more A-more B’ mapping between different dimensions, e.g. the bigger the trains are, the faster they are perceived. This relation, however, is not always true for both directions, i.e. one dimension can interfere with the other dimension, but not vice versa. Dormal et al. [20] did not find any interference effect of temporal cues on numerosity processing. Droit-Volet et al. [25] also showed no interference of duration with numerical discrimination in a numerical bisection task.

There is an ongoing debate on the brain areas involved in perception of numerosity and duration (time as a more general term). Imaging and brain stimulation, as well as lesion studies have tried to find the neural substrates involved in the perception of these two dimensions. The majority of studies have reported parietal regions presupposed (for a review see [26] and meta-analysis see [27]). Walsh [13], [14] proposed in a theory of magnitude (ATOM) the parietal cortex as the common brain area, involved in perception of time, space, number, size, speed and other magnitudes. Subsequently, Bueti and Walsh [28] revised this theory. ATOM revolves primarily around the role of the parietal cortex (as the major area for sensory integration and object manipulations), needed for active interactions with the environment in order to acquire knowledge. This theorem, however, does not fully explain how this area contributes to the cognition of magnitude in different dimensions. Contrary to Walsh [13], [14], Dormal et al [29] showed a contribution of frontal areas in decision-making in numerosity and duration processing.

Although there are many studies on time and numerosity perception, there are only a few studies looking at how perception of these two dimensions link to each other. Cappalletti et al. [30], in a lesion study, showed the dissociation between duration, numerosity and space processing. Using TMS, Dormal et al. [31] showed a similar effect. They demonstrated that the stimulation of left IPS impaired performance in a numerosity comparison task, whereas duration comparison was not affected. Only recently Dormal et al. [29], in a functional magnetic resonance imaging (fMRI) study, showed activation of the IPS and areas in the pre-central, middle and superior frontal gyri for both numerosity and duration processing. Moreover, based on psychophysiological interaction (PPI) analysis, they proposed that the right IPS contributes in both numerosity and duration processing.

**Figure 5:**
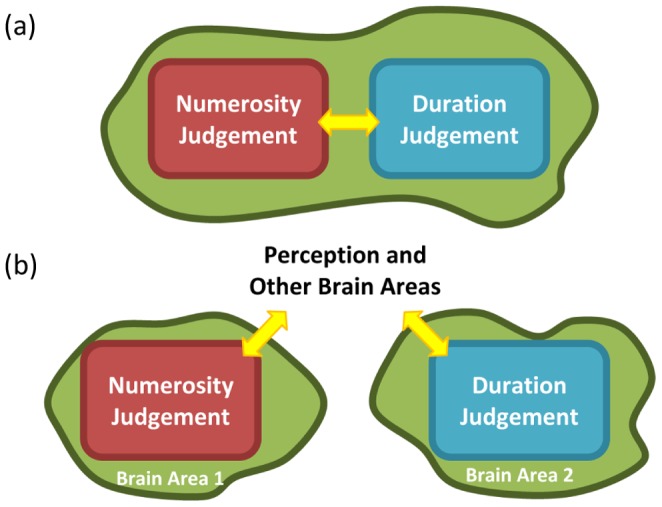
Two possible mechanisms explaining the effect of training on interference of numerosity on duration judgement. (a) based on the commonalities of numerosity and duration judgement, (b) through higher levels of cognition and other brain areas involved for numerosity and duration perception.

To the best of our knowledge, while there are many studies on interference of magnitudes in different dimensions (see above), none address the possible modulatory effect(s) of training on this interference. We trained participants on numerosity judgement and investigated whether their sharper perception of numerosity interferes more strongly with their perception of duration. The reported interference effect might result from two different mechanisms or a combination of the two. One possible mechanism stems from the commonality of numerosity and time perception in some brain areas (see above). As a result of training these brain areas may have been fine-tuned for more precise perception of numerosity, which consequently increased the interference of numerosity with perception of duration, see Figure 5(a). Another possible explanation is that the interfering effect of sharper perception of numerosity is mediated through higher levels of perception and/or other brain areas responsible for magnitude perception, i.e. the encoding of duration is intact but the integration of this coding with that of numerosity leads to higher interference, see Figure 5(b). Considering the vast evidence on brain areas involved in numerosity and duration perception, we propose that the combination of these two mechanisms underlies the increase in interference of numerosity in duration judgement. Further research needs to be done to differentiate between these two possibilities.

The ability of the brain to respond to training in a specific task by altering and adapting structurally as well as functionally has been investigated greatly in the literature (for a review see [32], [33]). These studies have led to an extensive body of evidence revealing lifelong plasticity. Nonetheless, the evidence on these changes is ambiguous. The follow-up study revealed that the effects of training fairly decreased over the 3 months post-training. Therefore, we speculate that the functional and possible structural effects of training were not long lasting. To our knowledge, no prior study has investigated the structural or functional changes of training in numerosity or duration judgement. Further research using imaging techniques could reveal not only whether training was sufficient to lead to changes within the brain, but would also allow more generalisable insights into the underlying neural structures of magnitude perception.

The control group did not undergo a training procedure as the training group did. Therefore, one might argue that the reported effect, i.e. training in numerosity judgement enhanced the interference of numerosity in duration judgement, is purely a result of difference in procedure. We, however, argue that the modulatory effect of training cannot be due to lack of training in the control group, as the only difference that we observed between control and training groups lied in their performance in phantom trials, reflected as steeper slope (increased sensitivity), and not in veridical trials, and neither in response times.

By increasing the number of items in each set and shortening the duration of presentation of each set, we aimed to match the difficulty of judgement in the two dimensions [21]. It has to be mentioned that the reported interference could be dependent on the magnitudes used in this study, e.g. decreasing the numerosity to a range that is easier to count (for example less than 10) might abolish the interference effect. Therefore, extending these findings to other magnitudes and dimensions (such as space or numeric symbols) needs careful considerations.

Another interpretation of results could be as follows: switching the task from numerosity judgement to duration judgement (for the training group) could indeed disturb the participants, due to the distribution of attention to two dimensions, leading to higher interference when compared to the control group. Results, however, showed that this is not the case, as the performance of the participants in the training group was comparable with that of control group for the veridical condition. In a more exaggerated way, it could be the case that participants based their decision on numerosity of the two sets in trials with equal presentation length. Based on the results, this possibility can be dismissed as well, as participants in the training group, especially, after such an intensive training did not achieve high performance percentage for phantom trials. This shows that participants kept their decision based on the duration of the two sets and they were not aware of the interference of numerosity of the two sets. Additionally, post study interviews revealed that even if participants detected the difference in numerosity of the two sets, they ignored the variation in that dimension.

On another note, the effect of training could turn out to be facilitatory, rather than interference. Based on this study, it cannot be determined whether this effect is found through reversed intervention, i.e. training in duration judgement and testing on numerosity judgement, and even if, whether it will go in the same direction, i.e. training duration perception may lead to facilitation, instead of interference in the judgments of numerosity. Further research needs to determine whether dimensions are interchangeable, so that training in one specific dimension leads to interference while another may lead to facilitation, or whether a hierarchy of dimensions exists, where one dimension takes a superior role over others.

We focused on investigating the dimensions duration and numerosity, while disregarding space, with the aim of dissociating these two dimensions. Further research should investigate the relationship between all three dimensions, which may allow insights into which dimension plays the predominant role in our judgments.

In conclusion, our results showed that training participants to more clearly perceive the difference in the number of items in the two sets affected their perception of duration presentation. Research on magnitude perception remains in its infancy and many more questions are yet to be answered. Future research needs to address the paramount question concerning specific operations underlying magnitude representation [28] and their interconnections.
